# Learning to practise the Guided Self‐Determination approach in type 2 diabetes in primary care: A qualitative pilot study

**DOI:** 10.1002/nop2.76

**Published:** 2017-02-10

**Authors:** Bjørg Oftedal, Beate‐Christin Hope Kolltveit, Vibeke Zoffmann, Åsa Hörnsten, Marit Graue

**Affiliations:** ^1^ Department of Health StudiesUniversity of StavangerStavangerNorway; ^2^ Centre for Evidence‐Based PracticeBergen University CollegeBergenNorway; ^3^ The Research Unit Women's and Children's HealthThe Juliane Marie CentreCopenhagen University HospitalCopenhagenDenmark; ^4^ Department of NursingUmeå UniversityUmeåSweden

## Abstract

**Aim:**

To describe how diabetes nurses in primary care experience the process of learning to practise the person‐centred counselling approach Guided Self‐Determination among adults with type 2 diabetes.

**Design:**

A descriptive qualitative design.

**Method:**

Data were collected in 2014–2015 by means of individual interviews with four diabetes nurses at two points in time. The data were analysed using qualitative content analysis.

**Results:**

Three themes that reflect nurses’ processes in learning to use the Guided Self‐Determination approach were identified: (1) from an unfamiliar interaction to “cracking the code”; (2) from an unspecific approach to a structured, reflective, but demanding approach; and (3) from a nurse‐centred to a patient‐centred approach. The overall findings indicate that the process of learning to practise Guided Self‐Determination increased the nurses’ counselling competence. Moreover, the nurses perceived the approach to be generally helpful, as it stimulated reflections about diabetes management and about their own counselling practices.

## 1 Introduction

Type 2 diabetes mellitus (T2DM) is undoubtedly one of the most important health challenges of the 21st century (IDF, 2015). At present, 382 million people worldwide are living with diabetes, a figure estimated to rise by 2035 to 592 million, of whom over 90% will have T2DM (IDF, 2015). T2DM is a chronic condition that involves daily, complex self‐management behaviours, such as diet, physical activity, blood glucose monitoring and sometimes medication, to achieve metabolic control and prevent long‐term complications (Cefalu, 2016; IDF, 2015). Previous research has shown that many people with T2DM find it difficult to self‐manage their diabetes condition, as its management requires considerable self‐discipline and motivation (Carolan, Holman, & Ferrari, 2015; Oftedal, Bru, & Karlsen, 2011). The International Diabetes Foundation (IDF) argues that without effective patient counselling methods in diabetes care, the burden of living with the disease will continue to increase (IDF, 2015). Obviously, there is a need to develop counselling methods and competences aimed at stimulating motivation for adequate diabetes management. The World Health Organization [WHO] (2013) emphasizes the importance of person‐centred care (PCC) to promote better health outcomes and improve well‐being. PCC refers to a philosophy that understands patients as equal partners in planning, developing and assessing care rather than focusing on the disease (de Silva, 2014; Olsson, Jakobsson Ung, Swedberg, & Ekman, 2013). From this perspective, PCC focuses on consultations where health professionals use counselling methods to activate and motivate person to become partners in healthcare decisions (Coulter et al., 2015; Olsson et al., 2013). The purpose of PCC is to provide support that is attentive and tailored to patients’ beliefs, values and preferences and to empower patients to improve and manage their own health (McCormack & McCance, 2010). Most literature to date has assumed that PCC is effective. However, a systematic review found that person‐centred care interventions were shown to be effective in 8 of 11 studies (Olsson et al., 2013). In diabetes care, PCC interventions have resulted in significantly decreased glycated haemoglobin (HbA1c) (Hörnsten, Lundman, Stenlund, & Sandström, 2005; Jutterström, 2013; Seitz, Rosemann, Gensichen, & Huber, 2011; Zoffmann, Vistisen, & Due‐Christensen, 2015) and emotional distress, as well as increased competence and motivation among patients (Hörnsten, Stenlund, Lundman, & Sandström, 2008; Zoffmann & Lauritzen, 2006; Zoffmann et al., 2015). However, a prerequisite for successful PCC in clinical practice is that nurses are competent counsellors who place the person at the centre of the care (Bergh, Persson, Karlsson, & Friberg, 2014; Friberg, Pilhammar Andersson, Bengtsson, & Andersson, 2007). Hence, training is essential (Friberg et al., 2007). A systematic review found that only 10 of 43 studies reported that healthcare providers were trained in PCC (Dwamena et al., 2012). Another study reported that although nurses undertook PCC education, they lacked sufficient support and training to be confident in practice (Boström, Isaksson, Lundman, Lehuluante, & Hörnsten, 2014). Consequently, the lack of formal counselling training led to frustration and ambivalence as well as reduced motivation for PCC among healthcare providers (Boström et al., 2014). Therefore, more studies are required to explore learning trajectories and counselling practices, to create realistic conditions that allow the intervention to be implemented in everyday work, particularly in primary care (Bergh et al., 2014). According to the International Diabetes Attitudes, Wishes and Needs (DAWN) study (Bootle & Skovlund, 2015), “the educational process is the key to success and promoting understanding” of PCC (p. 15). Thus, there is a need for studies investigating the qualification process for person‐centred supervisors in daily primary care (Bergh et al., 2014). In this study, we report on the process of learning to practise the person‐centred counselling approach, Guided Self‐Determination (GSD), among diabetes nurses (DNs) in primary care (Table 1).

**Table 1:** Presentation of the themes and sub‐themes of diabetes nurses process of learning to practice the GSD approach

Themes	Sub‐themes
From an unfamiliar interaction to “cracking the code”	Initially “groping in the dark”.
	Frequent repetition and feedback promotes confidence
From an unspecific approach to a structured, reflective, but demanding approach	Expectation of a successful tool and increased competence
	A distinct and focused method
	Time‐ and energy‐consuming but a good investment
From a nurse‐centred to a patient‐centred approach	Stimulate reflections and responsibility—“open new doors”.
	Decreased control and increased insight

### 1.1 Background

Guided Self‐Determination is a theory‐driven counselling approach founded in a synthesis of grounded theories (Zoffmann, Harder, & Kirkevold, 2008; Zoffmann & Kirkevold, 2005, 2007), self‐determination theory, life‐skills theory and humanistic values theory (Zoffmann et al., 2016). The method was developed and tested among adults with difficulties related to their type 1 diabetes to promote empowerment, decision‐making and motivation for diabetes management (Zoffmann, 2004). It consists of seven consultations using several structured reflection sheets. The development of reflection sheets was based on the driving theories and intended to empower the individual to become self‐determined and to develop life skills adequate to manage challenges in diabetes management (Zoffmann et al., 2016). The reflection sheets encompass four themes: the person–provider relationship, life with diabetes, the relationship between the ideal and reality and working to change. The purpose is to guide person and health professionals through mutual reflection (Zoffmann & Lauritzen, 2006) using a six‐stage interaction process: (i) establishment of a mutual person–nurse relationship with clear I‐you‐borders (ii) self‐exploration, (iii) self‐understanding, (iv) shared decision‐making, (v) action and (vi) feedback from action. At each consultation, the patient completed the reflection sheets in advance to stimulate reflections prior to and during consultations with DNs (Zoffmann & Kirkevold, 2012; Zoffmann et al., 2016). Reported effects in randomized controlled trials (RCT) show significant increases in perceived competence, autonomous motivation and quality of life, as well as decreased HbA1c, emotional distress and motivation among adults with type 1 diabetes (Zoffmann & Lauritzen, 2006; Zoffmann et al., 2015), but no effect was found among men (Zoffmann et al., 2015).

As GSD was originally designed for people with a type 1 diabetes, a project group consisting of user‐representatives of people with T2DM, researchers and nurses experienced in using the GSD, modified the GSD to suit people with T2DM. Through this modification process, the number of consultations was reduced from seven to four, making it more time‐efficient. In addition, reflection sheets were reduced in number from 21 ‐ 17 without losing the essentials of GSD, such as reflection on dynamic judgement building. The adapted GSD was completed before the DNs used the approach in consultations among people with T2DM.

However, although GSD is recommended and reveals positive health outcomes, few studies have explored how DNs experience the process of learning to use GSD. According to Jarvis (2015), learning is a complex process whereby the whole person experiences a social situation. This experience can be transformed by a combination of reflections, emotions and actions and always results in a changed—i.e. more experienced—person. The aim of the current study was therefore to describe how diabetes nurses in primary care experience the process of learning to practise the person‐centred counselling approach, Guided Self‐Determination, among adults with T2DM.

## 2 The study

### 2.1 Design

This pilot study has a descriptive qualitative design. The study was conducted over 5 months in 2014–2015 by means of individual interviews with four DNs in GP at two points in time.

### 2.2 Sample

A purposive sample of DNs was recruited from GP in primary care in southwestern Norway. To obtain a sample that consisted of nurses with particular diabetes expertise, the first author (BO) disseminated information about the study during a professional meeting for DNs and by telephone to GPs and invited them to participate. As many GPs in Norway did not have employed Registered Nurses, potential recruits for this study were few. However, the inclusion criteria were as follows: Registered Nurse employed in a GP, more than 1 year of experience in diabetes care and a willingness to participate in GSD training and to use the GSD method when counselling people with T2DM. The head of the GP approved their participation in the study. In total, four DNs working at different GPs consented to participate; all were women of ages 34–54 who had experience in diabetes care ranging from 2 to 20 years (mean = 8 years). Three of the four DNs had formal postgraduate education in diabetes care (60 ECTS).

### 2.3 Guided self‐determination training

The four DNs included in this study attended the Steno Diabetes Centre for training in the original GSD in 2014. The programme consists of four course days (24–32 hr) over 9 months and delivers competences in using GSD. It includes lectures in the theoretical foundation and application of the reflection sheets, workshops, discussions, supervisions and practising the use of GSD in own clinical practice. The participants were trained in three advanced professional communication skills: mirroring, active listening and values‐clarification response. As part of the clinical practice, each DN recruited two adults with T2DM from their GPs to participate in seven GSD consultations. At the end of the course, the DNs completed a test regarding the GSD approach. A validity assessment tool evaluated whether the DNs’ performances with GSD were congruent with its theoretical foundation. To acquire more experience in practising in GSD, each DN recruited two additional patients with T2DM from their GPs to participate in the GSD method adapted to T2DM, which consisted of four consultations and fewer reflection sheets. As part of this training, each DN participated in three group counselling guided by two GSD supervisors (V.Z. and J.M.).

### 2.4 Data collection

Data were collected over 5 months in 2014–2015 and individual interviews were performed at two points in time: (i) after the DNs had received training and gained some experience using the GSD during consultations with two adults with T2DM; and (ii) after the DNs had used the adapted GSD for two additional adults with T2DM. The rationale to conduct interviews at two points in time was to provide insights and understanding of the process of learning to practice GSD. Each interview took place at the university and each was 40–60 min in length. A semi‐structured interview guide consisting of open‐ended questions about DNs’ experiences of learning to practice the GSD was used in both interview sessions. The first session began with questions related to the expectations of the GSD training and progressed to questions specific to their practice: “Could you please tell me about your experiences in practising the method”? “What challenges did you experience”? and “Can you describe situations where you mastered the method”? The participants were asked to give concrete examples of their experiences. These questions were followed up in the interview guide during the second interview session.

### 2.5 Analysis

The individual interviews from the two points of time were combined for analysis to explore the process of learning over time. The individual interviews were analysed using the qualitative content analysis described by Graneheim and Lundman (2004). Qualitative content analysis is a process of interpreting manifest and latent content and focuses on identifying similarities and differences in texts. The analysis process consisted of several steps. First, the transcribed text from both interview sessions was read by two members of the research team (BO and MG) and meaning units responding to the aim were identified. The meaning units were condensed, with the core message retained. These were then labelled with codes (e.g. “difficult communication”), which were compared based on similarities and differences and consolidated into tentative sub‐themes (e.g. “demanding communication skills”, “more training is needed”) and themes (e.g. “demanding” but “cracking the code”). These sub‐themes and themes were discussed and refined in further analyses among the researchers (BO, ÅH, BCHK and MG) and presented in national and international conferences. Finally, three themes that described the sub‐themes were identified.

### 2.6 Rigour

We used the criteria of credibility, dependability and transferability to ensure the rigour of the research (Lincoln & Guba, 1985). In this study, the DNs were interviewed twice, which may strengthen the credibility of the data, as data collection at two points in time can lead to a deeper understanding of an issue. To reinforce the credibility of the data collection, all interviews were conducted by the same researcher (BCHK). In addition, the interpretation's credibility was ensured through discussion among the researchers. The dependability of the study was obtained by using the same interview guide for all interviews and the interviews were audiotaped and transcribed verbatim and imported into QSR International's NVivo 10 software. The transferability of our findings to another context was enhanced by using illustrative quotations from the data.

### 2.7 Ethics

The Norwegian Social Sciences Data Services approved the study (No. 39454). All respondents provided informed written consent before the individual interviews and were guaranteed confidentiality and the right to withdraw from the study at any time.

## 3 Results

The analysis resulted in the identification of three themes: (i) from an unfamiliar interaction to “cracking the code”; (ii) from an unspecific approach to a structured, reflective, but demanding approach; and (iii) from a nurse‐centred to patient‐centred approach. We used quotes from all nurses in the result section; however to ensure confidentiality, the quotations from each participants are not labelled in the text.

### 3.1 From an unfamiliar interaction to “cracking the code”

This theme demonstrates that all DNs were initially unfamiliar with GSD, particularly with the communication skills, but frequent repetition and feedback from patients promoted confidence and belief in their ability to “crack the GSD code”.

#### 3.1.1 Initially “groping in the dark”

All DNs emphasized that GSD training at the Steno Diabetes Centre facilitated their understanding of how to use the reflection sheets and practise advanced professional communication skills. However, although the DNs had some training, they initially experienced using the method as “groping in the dark” and felt unfamiliar with the communication techniques as described by one nurse:

I feel this way of communicating is awkward. I feel mirroring is unnatural. It can perhaps become internalised, as it should be, but the method requires experience.

In addition to the reported difficulty in incorporating the communication skills, all DNs also experienced challenges in learning to use the reflection sheets. One nurses stated:

Working with the reflection sheets feels quite awkward. Especially in the beginning, I fumbled a bit and it took a lot of focus from the talk with the patient. I thought that was difficult and I did not know quite how to explain the forms [to the patient].

#### 3.1.2 Frequent repetition and feedback promotes confidence

The DNs reported that it became gradually easier to use GSD; however, they emphasized that they needed frequent repetition before becoming comfortable with it. In addition, patient feedback was an important factor in influencing their self‐confidence in practising the method, as they perceived this as confirmation that they were on their way to “cracking the code”. Moreover, feedback from the patients stimulated the nurses’ willingness to persevere in the process of learning to practise the method. One nurse reported:

I experience that the patients feel cared for and that they are very happy when we talk. I feel that something is achieved. There is a feeling of mastery when they leave and are happy.

### 3.2 From an unspecified approach to a structured, reflective, but demanding approach

This theme reflects that DNs experienced a shift from a diabetes approach developed from various sources and concepts to a structured approach characterized as a tangible tool, but time‐ and energy‐consuming. Overall, they perceived GSD as a good investment.

#### 3.2.1 Expectation of a successful tool and increased competence

The DNs reported that before they were introduced to GSD, their approach to diabetes was inspired by advice and ideas acquired from diabetes courses, seminars and conferences. One nurse reported:

I picked out what I think are good ideas and then I have done it my way, which sometimes works and sometimes does not.

However, although the DNs were not dissatisfied with their approach, they said that they sometimes failed or were unable to stimulate patients to achieve adequate diabetes management. They were, therefore, interested in learning a new way of counselling. Several DNs explained that they “wanted a tool to use” in the consultations.

Another reason for participating was that the DNs wanted to develop themselves as supervisors in the field of diabetes. Succeeding as counsellors was important for all DNs and throughout the training in and applying the GSD, they experienced improved communication skills and increased awareness of and reflections on their own counselling practices as exemplified by the following quotes:

It led to an awareness in me of a totally different way of communicating with the patient.

#### 3.2.2 A distinct and focused method

All DNs experienced the GSD as a tangible tool that allowed the dialogues with patients to be more focused, directed and structured. In particular, they perceived the reflection sheets as useful in achieving a more “to the point” conversation with patients. One DN explained:

Normally, we are not used to having anything defined or written in advance. Now you have something in writing—something concrete, which works as a starting point for each consultation. I think that is good.

The DNs reported that they saw the benefits of each individual working with the reflection sheets at home, as they came prepared to the consultations and had developed their own thoughts and reflections about their diabetes management:

Actually having something concrete [a sheet] to fill in for every consultation—and have your patient reflect on this at home; the various questions, their turning up prepared having thought some questions through and their being followed up and seen by the same person [is good].

Consequently, when patients came to consultations prepared and presented their own thoughts about their actual problems in diabetes management, DNs had better opportunities to respond to the challenges and to guide patients to find solutions that matched their own values.

#### 3.2.3 Time‐ and energy‐consuming but a good investment

It emerged from the first point in time that all DNs experienced the GSD, with seven consultations, as time‐ and energy‐consuming. This was also emphasized in the second occasion, when the DNs had used the modified GSD, which consisted of four consultations and fewer reflection sheets. Indeed, the DNs did not perceive the modified version as reduced, merely as more condensed, as it resulted in a more intensive consultation session. Accordingly, all DNs reported that the GSD method was still demanding and energy‐consuming and that they had to be completely aware in each consultation:

It takes time. You have to make time for it. And it requires that you are really “there” yourself. In order for me to feel that I provide a good GSD consultation, I have to be absolutely, one hundred per cent present.

However, despite perceiving the method as time‐ and energy‐consuming, all DNs emphasized that using GSD in their consultations was a good investment, as they felt they succeeded in stimulating patients’ reflections on and motivations for diabetes management:

I enjoy when you see that the patients think it is fun—when you see them offer up in their reflection sheets and you see that they have spent time on it. So I enjoy spending time on this [method].

### 3.3 From a nurse‐centred to a patient‐centred approach

This theme demonstrates shifting from an approach where DNs direct the consultation and “do things to” patients to patients directing their own care and working together with DNs to develop appropriate solutions. The sub‐themes describe what the nurses experienced and characterize their interaction with patients when using the GSD approach.

#### 3.3.1 Stimulate reflections and responsibility—“open new doors”

The DNs became aware that they had reoriented their support from the “compliance‐expecting” approach to a “user‐focused” approach to facilitate patients’ reflections, decision‐making, choice and autonomy:

We used to give advice all the time. If they said “I wish I could get more exercise”, I would say “you can do this or that”. Now, it is a more open way [of communicating], giving the patients room to figure out for themselves what is good for them. It sort of turns it around.

The DNs realized that using GSD changed the nature of interactions with patients, as GSD encouraged them to focus on understanding patients’ perspectives and priorities rather than quickly prescribing a standard treatment pathway. The DNs characterized the new GSD interaction as a positive and exciting process. One DN reported:

It is exciting to observe that it is a process—that the patient reaches a decision regarding changes—they become aware—the patient taking responsibility himself.

The DNs moved from feeling completely responsible for delivering adequate diabetes advice and information to focusing on stimulating patients’ responsibility for their own health. They said that it was easier to stimulate change in diabetes management when patients defined their own problems and solutions. Indeed, they perceived that it was essential to guide patients to identify problems and develop solutions in their own diabetes management:

That is the real goal—the patient sort of finding his own solution and gradually the solution to a problem as defined by himself. Then it is easier to make changes than if we only tell them how things should be done.

#### 3.3.2 Decreased control and increased insight

Another aspect that characterized the GSD interaction was that DNs said that they lost control as patients set the agenda in consultations. Consequently, one DN reported that she had to be “prepared for the unexpected”:

I'm the one in charge at annual check‐ups while, in these consultations, it is the patient who is in charge.

The DNs emphasized that they did not experience losing control as difficult. Rather, they explained this situation as a sensitive meeting with the patients, which gave them deeper insight into patients’ thoughts and experiences, as exemplified by one DN:

In a way, I'm intruding in their lives—their most intimate and vulnerable parts of themselves. It is quite personal. It isn't difficult, but it is still very sensitive. You have to tread carefully. But it isn't hard, it is just that I have to feel my way.

The willingness of many patients to share their vulnerable and deepest thoughts and narratives with the DNs affected the nurses strongly. Understanding how difficult it was to tell these stories, they were impressed by patients’ openness and honesty:

I think about how much the patient actually gives of him‐/herself when they respond to these sheets. They are supposed to draw metaphors and a picture. One patient wrote a long story, which was really touching to read. Just think of what it cost him to share so openly on paper. It made a deep impact really. It was amazing.

The DNs thought that without the GSD method, they were not sure that they would be able to capture these narratives, nor that the patients would have the opportunity to share these perspectives with them:

I don't think the patient could have shared this directly in words, how he experienced living with diabetes. You get a bit deeper when they have to think and reflect—and he opened up for a lot of topics that I might not have thought of asking him about.

## 4 Discussion

The aim of this study was to describe how diabetes nurses experience the process of learning to practise the Guided Self‐Determination counselling approach among adults with T2DM. Three themes were identified from the analysis of the individual interviews, all of which reflect the DNs’ process of learning to practise the method. According to Jarvis (2015), learning is about both conscious and unconscious experiences. Usually, these are simultaneous processes, but we can never be fully aware of the extent of either. However, learning occurs from the situations we experience and reflecting on how a situation appears to others allows an individual to experience other, perhaps new, aspects of a situation (Borell & Eriksson, 2013; Jarvis, 2015). In the current study, the analysis indicates that DNs in their learning process compared their earlier experiences in diabetes care with the new GSD approach and, in turn, reflected on the advantages and disadvantages of each. The analysis revealed a substantial agreement among the nurses about their negative and positive experiences with GSD. A possible interpretation may be that the DNs during the learning programme received group counselling, resulting in a uniform perception of GSD. We assume that a larger sample size would generate a more nuanced picture of the learning process.

Not surprisingly, the first theme, ‘from an unfamiliar interaction to initially “cracking the code”’, indicates that the learning process was initially difficult, as it required new ways of thinking, acting and communicating. The DNs were unfamiliar with communication skills such as active listening and mirroring. Person‐centred approaches such as GSD are typically not easy to master (Hope Kolltveit, Graue, Zoffmann, & Gjengedal, 2014; Jansink et al., 2013). WHO has, therefore, argued that counselling should be a lifelong‐learning approach, developed and refined over one's clinical practice (WHO, 2013). Findings seem to support such arguments, as DNs reported that they needed a lot of training before they felt confident in using the method. Moreover, findings indicate that positive feedback from patients stimulated and increased their self‐efficacy for “cracking the code”. The self‐efficacy component has been recognized as having important motivational effects on behaviours, though feedback and support from others are external motivational factors associated with less optimal motivation for behaviour change. According to Ryan and Deci (2000), there is a continuum from extrinsic to intrinsic motivation and the former can lead to the latter. Therefore, it is plausible to suggest that feedback from patients is critical in stimulating the newly trained DNs’ motivation for performing and continuing with GSD. Lindhe Söderlund (2010) found that newly trained healthcare providers are more in need of feedback from patients than healthcare providers who have practised longer and have more confidence in their counselling abilities.

The second theme, ‘from an unspecific approach to a structured, reflective, but demanding approach’, reflects that despite the new approach being perceived as more demanding than conventional diabetes care, the DNs discerned that GSD offered a structured approach that stimulated reflection. They reported that one reason for learning GSD was to be able to offer patients a tangible tool that stimulates better diabetes management. Another reason was that the DNs perceived the training as an opportunity to increase counselling competence and enhance their own development as supervisors. Several studies have demonstrated that nurses wish to help patients while at the same time realizing their own potential as nurses (Fealy, 2004; Kristoffersen, 2013; Tveit, 2008). Kristoffersen and Friberg (2015) argue that such ideas are not contradictory and may be prerequisite to managing today's complex and demanding clinical practices. She emphasizes that nurses who have the drive to develop themselves will grow and continue in nursing practice. It is a fact that, although the DNs reported that the GSD was demanding, none of them dropped out during the training, which may indicate that succeeding as counsellors was an important motivation. In addition, all DNs reported increased counselling competence. This finding is in accordance with another study (Juul, Maindal, Zoffmann, Frydenberg, & Sandbaek, 2014), which reported that most nurses who completed a GSD course perceived that they improved their communication skills and competence in autonomy support. However, as with other research in PCC (Boström et al., 2014; Jansink, Braspenning, Van Der Weijden, Elwyn, & Grol, 2010; Kääriäinen & Kyngäs, 2010; Mulder, Lokhorst, Rutten, & van Woerkum, 2015), the current study finds that the counselling method is time‐ and energy‐consuming. This brings us to reflect on the possibility of counselling more effectively. Researchers who investigated Internet‐based cognitive behavioural therapy (ICBT) found that ICBT is more time‐effective compared with conventional face‐to‐face CBT (Hedman et al., 2013), due to less therapy time being required in ICBT. Whether this is the case for GSD is yet to be studied.

The last theme, ‘from a nurse‐centred to a person‐centred approach’, shows that the features of the GSD method that the DNs considered significant were shaped by earlier experiences. In contrast to earlier consultations, where they felt entirely responsible for their patients’ diabetes management, the GSD method helped them to put patients at the centre of the care and to stimulate patients’ responsibility for their own health. Our findings indicate that the DNs appreciated that the GSD method allowed them to listen more and give patients opportunities to become more active and responsible in their diabetes management, even though this meant losing control over the direction of the consultation. This finding contrasts with research that revealed that DNs struggled with losing control of and responsibility for patients’ diabetes management (Boström et al., 2014; Hope Kolltveit et al., 2014; Hörnsten, Lindahl, Persson, & Edvardsson, 2014). One explanation for our findings could be that the majority of DNs in this study have had formal postgraduate education in diabetes care, which includes lectures in PCC (Graue, Rasmussen, Iversen, & Dunning, 2015) and that this has enabled the nurses to provide more PCC and to work in partnership with people with diabetes (Graue et al., 2015). Another explanation relates to the reflection sheets. A previous study reported that these helped nurses to navigate the consultations (Hope Kolltveit et al., 2014). Therefore, it is plausible that the reflection sheets may help nurses overcome their resistance to losing control, as they provide direction and work as a starting point for communication.

Consistent with another study in the field of PCC (Boström et al., 2014), our findings indicate that DNs using GSD experienced enriched relationships with patients. By giving patients opportunities to talk about the areas they found difficult, the DNs obtained deeper insights into patients’ vulnerabilities and strengths. Lindhe Söderlund (2010) highlight that a fundamental principle in PCC is communicating with patients based on what nurses know about them as people and developing a clear picture of what patients value in their lives. It may, therefore, be that through the process of learning to practise GSD, DNs have developed competence to conduct a person‐centred approach. Our findings are in accordance with other studies that found that it is possible to train DNs in PCC (Boström et al., 2014; Jutterström, 2013).

### 4.1 Limitations

In this study, we used a purposive sample and three out of four DNs had formal postgraduate diabetes education. A possible limitation could therefore be that these participants might have become more motivated for GSD simply because they had already reached an advanced level in diabetes care. Moreover, it is possible that the nurse without formal diabetes education was influenced by the other nurses’ motivation for GSD or experienced a form of peer pressure, which could be understood as a limitation in the current study. While we aimed for rich and varied data by using individual interviews at two points in time, the findings revealed a uniform perception of GSD among the nurses, which may limit the transferring of the ability to transfer these findings to other settings. Moreover, although the GSD training programme was structured and the nurses passed a test after completing the training, it is unclear how the DNs actually used GSD in consultations. Data from patients and course leaders or direct observation of skills using voice recordings could have enhanced our understanding of how the nurses practised GSD.

## 5 Conclusion

This study has contributed to knowledge about how DNs in GP experience the process of learning to practise the GSD counselling approach among adults with T2DM. The overall results indicate that DNs experienced GSD as a constructive counselling method in stimulating patients’ reflections and motivation for diabetes management. Moreover, the findings suggest that DNs perceived increased counselling competence and reflection about their own communication skills. In addition, by practising GSD, the DNs obtained deeper insights into patients’ strengths and vulnerabilities. However, findings also highlight that GSD is time‐ and energy‐consuming. This study has implications for clinical practice, education and research. First, as advanced communication is demanding and requires frequent repetition, a communication skills module should be available for all the nurses practising GSD on a yearly basis. Second, when implementing GSD in clinical practice, leaders should organize formal training in groups to increase nurses’ counselling competence. Lastly, further research with a larger sample size may enhance the relevance of the findings in this study.

## Conflicts of interest

We declare no conflicts of interest.

## Author contributions

BO, MG and VZ designed the study; BO and BCHK involved in data collection; BO, MG, BCHK and ÅH analysed the data; BO has mainly drafted the manuscript. All authors contributed to editing of the final manuscript, revised it critically for scientific content, read and approval the final version.

## Ethical considerations

The Norwegian Social Sciences Data Services approved the study (No. 39454). All respondents provided informed written consent before the individual interviews and were guaranteed confidentiality and the right to withdraw from the study at any time.
